# Mixed methods systematic review of the literature base exploring working alliance in the chiropractic profession

**DOI:** 10.1186/s12998-022-00442-4

**Published:** 2022-09-02

**Authors:** Dima Ivanova, Felicity L. Bishop, Dave Newell, Jonathan Field, Madeleine Walsh

**Affiliations:** 1 University of Southampton, University Road, Southampton, SO17 1BJ Hampshire UK; 2 Anglo European College of Chiropractic University College, Parkwood Campus, Parkwood Road, Bournemouth, BH5 2DF Dorset UK

**Keywords:** Working alliance, Chiropractor–patient relationship, Trust, Collaboration, Shared decision-making, Systematic review, Communication, Narrative synthesis, Thematic synthesis, Contextual factors

## Abstract

**Background:**

The construct of working alliance has been used to operationalise the patient–clinician relationship. Research evidence from the rehabilitation literature has established an association between the construct and several patient outcomes. The aim of this systematic literature review was to study working alliance in the chiropractic discipline.

**Method:**

This review followed a mixed method systematic review methodology: EBSCO (The Allied and Complementary Medicine Database), EBSCO (MEDLINE), EBSCO PsycINFO, Web of Science Core Collection, Chiro index, and grey literature were searched for quantitative, qualitative, and mixed methods studies on 17th March 2021. Qualitative appraisal was conducted using the Mixed Methods Appraisal Tool, version 2018. The qualitative component was synthesised via thematic synthesis and explored patients’ and chiropractors’ perceptions of the nature and role of working alliance. The quantitative component was synthesised via narrative synthesis to examine how the construct has been measured in research and what its effect on clinical outcomes and patient satisfaction is. The findings were integrated in the discussion section.

**Results:**

Thirty studies were included. The qualitative component found that both patients and chiropractors consider working alliance as a key factor in the treatment journey. The findings illustrated that the construct includes the bond between a patient and a chiropractor which is underpinned by trust and attentiveness to patients’ needs, values and preferences. Qualitative data also suggested that strong working alliance has the potential to improve patients’ adherence to treatment and that it is characterised by ongoing negotiation of expectations about the goals of care and the tasks involved in the treatment plan. The quantitative component highlighted that even though working alliance is relevant to the chiropractic discipline, very few studies have quantitatively measured the construct and its effect.

**Conclusion:**

The findings of this review emphasise the subjective importance of working alliance in the chiropractic clinical encounter. However, there were not enough homogenous studies measuring the effect of working alliance on clinical outcomes and patient satisfaction to conduct a meta-analysis. Future research should focus on evaluating potential direct and mediated effects on patient outcomes.

**Supplementary Information:**

The online version contains supplementary material available at 10.1186/s12998-022-00442-4.

## Methods

### Introduction

In many clinical encounters the targeted treatment regime alone is unlikely to fully explain patients’ clinical outcomes [1]; the relationship between the patient and the clinician is also a critical component [2]. Working alliance (WA) is a construct that has been used to operationalise this professional relationship [3]. WA incorporates cognitive and emotional dimensions of the interpersonal processes between both parties occurring during care [4]. Research has demonstrated that WA is associated with physical function, pain, disability, patient satisfaction, adherence to the treatment plan and overall perceived effect of treatment [5–9].

The term WA originated from psychotherapy and there is uncertainty around its conceptualisation [4, 10]. This review adopted Bordin’s [11] formulation because it claims to be universally applicable [10–12]. According to Bordin [11], a person and a therapist, or in this case a chiropractor, unite against a common foe, for example, chronic low back pain, and work towards a common goal, such as improved physical function [10, 11]. A strong WA between the individual who strives for a change and the person who helps them (the change agent), is crucial for the change process itself and requires ongoing negotiation of expectations [11]. WA has three key features: shared decision making and agreement on goals of the change process, collaboration on the tasks required to achieve these goals, and establishment of a bond which is based on reciprocal feelings of liking [3, 11]. The mutual bond embraces interpersonal processes such as trust, acceptance and confidence and is often conceptualised in the literature in relation to patient’s perception of the therapist’s empathy [10, 13]. A systematic review of randomised control trials and cohort studies suggested that patients’ perception of the quality of the WA during treatment is a predictor for improved physical functioning and reduced pain in patients with chronic musculoskeletal pain: authors consequently recommended that practitioners should be sensitive to and enquire about patients’ perceptions of the WA [14].

Historically, chiropractors have identified themselves with a treatment predominantly focused on spinal manipulation. Increasingly however, evidence suggests that the idea of spinal manipulation being the single cause of observed clinical outcomes is unfounded given the evidential impact of contextual factors, which are part of all clinical encounters [15, 16]. Patients’ interpretation of these factors amongst which are interpersonal processes inherent in WA can trigger contextual effects through innate neurophysiological mechanisms and thus impact clinical outcomes [15]. Furthermore, it could be argued that strong WA can provide the foundational environment necessary for patients to benefit from the effects of contextual factors present in all chiropractic consultations [17] by eliciting psychological and/or behavioural changes [18]. For example, a large prospective cohort study illustrated that WA decreased disability at least partly by improving patients’ self-efficacy for coping and reducing psychosocial distress and the perceived threat of low back pain [5]. A more comprehensive understanding of WA will enable chiropractors to learn how to skilfully use contextual factors which in turn can drive modulation of pain [15].

To our knowledge, a review of the evidence base regarding the WA between a patient and a chiropractor has not been conducted. Consequently, this mixed methods systematic review aimed to synthesise qualitative and quantitative evidence to study the nature and the role of WA within chiropractic consultations. The qualitative component of this review identified and synthesised literature concerning patients’ and chiropractors’ perspective on the construct. The quantitative component reviewed additional literature investigating how WA and its features have been measured in the chiropractic literature and the effects of WA on clinical outcomes and patient satisfaction.

### Design

This review followed mixed method systematic review methodology [19, 20] to consider a diverse body of information exploring WA in chiropractic and, ultimately, to inform evidence-based practice [21]. The review used a convergent segregated approach to synthesis and integration [20, 21]; the review comprises a qualitative component, a quantitative component, and an integrative interpretation of both components (the latter forms the basis of the Discussion section of this article). Separate quantitative and qualitative syntheses were conducted in parallel before the reviewer then integrated the findings from both syntheses to develop a more comprehensive interpretation. It was expected that the data and results from the quantitative studies, together with the quantitative component from the mixed methods studies, would complement the data and results from their qualitative counterparts [20]. The protocol for this review was registered with PROSPERO on 17.03.2021 (CRD4202123809) and can be accessed online [22]. The review has been reported in accordance with Preferred Reporting Items for Systematic Reviews and Meta-analyses (PRISMA) statement [23].

### Eligibility criteria

#### Types of studies

This review included quantitative, qualitative, and mixed methods studies. The qualitative component of this review considered all studies that identify and explore the perceptions and experiences of patients and their chiropractors. For studies with a broader focus, only the data relevant to WA were extracted. There is a diversity of available measures of WA in the literature, which possibly reflects the ambiguity around the conceptualisation of the term [4]. Therefore, the quantitative component focused on studies that measured WA either implicitly or explicitly. In other words, measures which are not specifically designed to measure WA were also considered if they assess a construct related to Bordin’s [11] formulation of WA and its three features. Only articles with full text in English were included. No filters were applied for publication dates. Table 1 illustrates how the Population, Phenomena of Interest, Context (PICo) mnemonic guided the eligibility criteria [24].

**Table 1:** Eligibility criteria

Component	Inclusion and exclusion criteria	Rationale
Population	Studies that include chiropractors and their patients. The inclusion criteria for chiropractors were to be licensed practitioners. If the study involved data collected from other healthcare providers or stakeholders, the study was included only if the data collected from eligible participants could be differentiated from the rest	To study working alliance (WA) in the context of the chiropractor–patient relationship
Phenomena of interest	Studies which have either explored WA between a patient and a chiropractor or have implicitly or explicitly explored one or more of the three features of WA proposed by Bordin [11]: agreement on goals, agreement and collaboration on the tasks required to achieve these goals and the establishment of a bond	To synthesise all findings relevant to the research questions, including the different constructs that relate to WA and any information relevant to the conceptualisation of WA in the context of the profession
Context	The eligibility criteria were not bound to a specific country, ethnicity, or settings, but excluded studies exploring the WA in the context of physiotherapy, osteopathy, or alternative and complementary therapies other than chiropractic	To study WA between a patient and a chiropractor in different contexts

### Search strategy

The search strategy used the databases which were considered by the multidisciplinary team of reviewers as relevant to the research question and aimed to locate both published and unpublished studies.

#### Information sources

EBSCO (AMED—The Allied and Complementary Medicine Database), EBSCO (MEDLINE), EBSCO PsycINFO, Web of Science Core Collection, Chiro index were searched for potentially eligible articles on 17th March 2021. The lead reviewer screened the references of the articles selected for critical appraisal. Grey literature considered for the review included, for example, conference abstracts, unpublished trial data, theses found in sources such as Google Scholar, NHS Evidence, Trip (Turning Research into Practice) database, EThOS and OpenGrey.

#### Search terms

The search strategy included terms for WA, its features, and chiropractic, combined using Boolean operators. Additional search strategy information is available in the protocol [25]. Table 2 shows the search terms and how they were combined.

**Table 2:** Search terms

(Boolean operator) Key word	Search terms
Working Alliance	“doctor–patient relation*”, “physician–patient relation*”, “patient–therapist relation*”, “patient–therapist relationship*”, “practitioner–patient relationship*”, “therapeutic relationship*”, “therapeutic relation*”, WA*, “helping WA*”, “working allience*”, “therapeutic WA*”;
(OR) Agreement on tasks and goals	“shared decision making”, “decision making”, agree*, “individuali*ed care”, “person centred care", “person centered care”, “goal setting”, “setting goals”, goal*, collaborat*;
(OR) Bond	bond*, “mutual liking”, trust, empathy, empathetic, confidence, appreciation;
(AND) Chiropractic	chiropractic, chiropractor*

#### Selection process

Two reviewers screened titles and abstracts independently using Rayyan [26]. Screened, potential eligible studies were then read in full to confirm their eligibility. Differences in opinion were discussed and resolved. Some citations from the databases were from chiropractic conference summaries containing multiple abstracts; each such abstract was screened manually by one reviewer. Lastly, one reviewer also screened the reference list of the already included articles.

### Data collection process

The reviewer collected the following data from all papers: study aims, participants, methodology, methods of data collection, methods of analysis, key results, and details about the research context, and information about the conceptualisation of WA or its three features.

### Quality appraisal

The quality appraisal was conducted by two reviewers using Mixed Methods Appraisal Tool, version 2018 (MMAT), which includes a combination of individual components and mixed method approaches [27]. MMAT focuses on assessing the methodological quality of the studies as the most essential criteria when it comes to validity of the findings [27]. The evaluation of methodological quality in this review ensured transparency about the limitations of the papers, even though studies were included based on relevance to the research question rather than MMAT score [27].

### Qualitative synthesis

Qualitative studies, and mixed methods studies that included qualitative component were subjected to a thematic synthesis: qualitative data were extracted and grouped into themes [28] to explore the nature of WA and patients’ and chiropractors’ perception of it. The qualitative component consisted of the “results” or “findings” section of the studies, including quotes and the authors’ interpretations of their qualitative data. The synthesis followed the three phases described by Thomas and Harden [28]: coding, developing descriptive themes, and generating analytical themes. The synthesis started with a focus on the data with free line-by-line coding during the first phase which enabled familiarisation with the data. The subsequent readings ensured that information relevant to WA was coded according to its meaning and content. The second phase involved grouping the identified codes into descriptive themes and comparing the initial codes across studies, merging some of them and creating new ones. The third phase involved generating analytical themes to produce a framework exploring the nature of WA in chiropractic consultations [28]. During the third phase of the synthesis, the qualitative data were reviewed in a deductive manner to look for codes that explore WA from the perspectives of chiropractors and patients. During the last two phases, the qualitative synthesis was additionally reviewed by and discussed with F.L.B. (an experienced qualitative researcher), D.N., and J.F. to ensure coherence between the generated themes and the corresponding qualitative data.

### Quantitative synthesis

Narrative synthesis was chosen instead of meta-analysis because of the clinical and methodological diversity of the ways that WA and its features were explored [21, 29]. The narrative synthesis aimed to examine how WA and its features have been measured in the literature and what the impact of WA is on clinical outcomes and patients’ satisfaction [30]. To address these questions, firstly, data was extracted from the primary studies in tabular form to generate a preliminary synthesis of findings. Then, the data was explored to determine whether studies could be clustered according to the characteristics in the data-extraction table [30]. For example, the data was clustered and grouped depending on the measurement tool used to study WA. Next, the relationships in the data were examined to produce a narrative synthesis via tabulation illustrating how WA or its features [11] were measured. A similar process was used to show the effect of WA on clinical outcomes and patients’ satisfaction.

### Integration of quantitative component and qualitative component

The convergent segregated approach to integration was selected because the qualitative and quantitative research components were expected to address different dimensions of WA [21]. The convergent design enabled the comparison of qualitative and quantitative findings [31]. The results of the qualitative and the quantitative synthesis were configured according to the Joanna Briggs Institute (JBI) methodology for mixed methods systematic reviews [21]. This involved quantitative components and qualitative components being configured to explore if individual syntheses were supportive or contradictory, if the qualitative findings explained the quantitative findings, and to find out if all parts of the quantitative component were explored in the qualitative components and if all parts of the qualitative components were tested in the quantitative components [21].

## Results

### Study inclusion

The database search resulted in 3913 records, of which 1597 were duplicates. The remaining 2315 records were screened. Citations of conference summaries contained 849 additional abstracts and were screened separately. Citation searching included the screening of 1287 references. The PRISMA flow diagram illustrates the study selection process [23] and is available as Additional file 1: Figure S1.

### Quality appraisal

Sixteen quantitative, seven qualitative and seven mixed method studies were reviewed after passing the screening criteria of the MMAT [27]. Additional file 2: Table S1, shows MMAT scores for the design criteria: each included study was assessed based on five questions (presented in the table captions) depending on its methodology. For mixed-method studies, quality appraisal was first completed against the mixed method design criteria. Then the qualitative and quantitative components were assessed separately. The most common concern in the quantitative studies was the risk of nonresponse bias, a challenge reported from previous research projects exploring the chiropractic profession [32, 33]. While qualitative studies were appraised highly on the methodology criteria, the qualitative components of some mixed methods studies were not reported in sufficient details [34–36].

### Qualitative component

#### Summary of included studies

The included qualitative studies and qualitative components of mixed methods studies used observations, interviews, and focus groups. The qualitative findings of two mixed-method studies were reported in limited details, hence they did not contribute substantially to the thematic synthesis [34, 36]. Table 3, which is located at the end of the document text file, summarises study characteristics.

**Table 3:** Summary of studies included in the qualitative synthesis

Study reference number	Country	Participants	Data collection methods	Data analysis methods
[37]	United States (US)	Trained and licensed chiropractor in a small town in the American Midwest	Ethnographic case-report	Not explicitly specified, but healing performance of a chiropractor is proposed to contain four intrinsic claims to trustworthiness
[34]	US	20 Patients at the group Health Cooperative of Puget Sound	Discussion groups	Information for the qualitative analysis and findings was limited
[44]	Canada	6 Chiropractors licensed with the College of Chiropractors of British Columbia	Interviews	Thematic analysis
[42]	US	15 Randomly selected participants from the 29 participants randomised to the chiropractic group	Interviews	Grounded theory approach
[46]	Canada	197 Participants were recruited from 20 participating chiropractors in Ontario	Interviews using Flanagan’s Critical Incident Technique	Inductive content analysis
[35]	Australia	208 Patients were observed	Recording duration of all patient–practitioner interactions was recorded, some were audiotaped, notes taking	Thematic analysis
[36]	Australia	9 Chiropractors and 173 patients	Interviews	Data were analysed by comparing the responses of individual patients with those of their practitioner in each of 173 case studies
[39]	US	171 Participants part of a randomised control trial	Interviews	Interactive approach to qualitative content analysis
[45]	Canada	6 Focus groups, a total of 69 patients	Focus group sessions	Qualitative content analysis (an interpretive approach)
[38]	US	A male family chiropractor and a sample of 57 people, who made a total of 104 office visits between them	Data were collected through (1) audiotape of all clinical interaction of the chiropractor for 8 days, (2) formal and informal interviews with the chiropractors., his staff, and patients	Data was content analysed using the modified Bales method of process analysis [69]
[43]	Canada	11 Chiropractors and nine patients	Interviews	Grounded theory approach
[47]	US	60 Participants in the Crotched Mountain	Individual interviews or focus groups	Thematic content analysis
[40]	Canada	3 Female patients and 3 male patients in the Halifax metro area, Nova Scotia, Canada	Focused ethnographic approach involving 16 semi-structured interviews	A systematic approach for analysing ethnographic data developed by Roper and Shapira [70]
[41]	Canada	90 Participants were recruited from two private chiropractic clinics in Calgary, Alberta, Canada	Interviews	Thematic analysis

#### Themes

The thematic synthesis generated five themes: (1) Chiropractic care as a change process; (2) Chiropractic treatment as collaboration; (3) Communication; (4) Patient-centredness as agreement on values, preferences and needs; and (5) Trust. The theme (3) Communication consists of two subthemes: Effective communication and Conflicts. Each of the themes is explained and illustrated by example quotes.

### Chiropractic care as a change process

This theme demonstrates how the qualitative findings portrayed chiropractic care as a change process. In an ethnographic case report, Bolton [37] analysed the therapeutic encounters between one chiropractor and his patients as “*a communicative and performative event*” (p. 309). The author [37] proposed that during each encounter a “*healer*” is expected to validate four intrinsic claims which are generalisable to different therapeutic approaches and which can be validated in diverse ways depending on the “*healer*” (p. 309). A chiropractor is expected to validate each claim they elucidate to establish and maintain trust between them and their patients [37], and one of these claims states “*I am making changes that will be realised in an improvement in your illness*” (p. 309). Applying Bordin’s [11] formulation of WA to Bolton’s analysis [37], the role of the chiropractor in patients’ care could be viewed as that of a change agent. In another study exploring the process of establishing trust, Oths [38] described how a chiropractor tends to explain to new patients that this change may be a prolonged process: “*Most people don't feel better 'til after several treatments. Be patient, don't get discouraged. It takes time.”* (p. 96). While in general the passage of time is necessary for the validation of the claim that a practitioner will bring change to patients’ circumstances [37], “*the patient is invited to accept or autonomously chooses to accept other more immediate criteria by which to validate it*” (p. 315). For example, change is “*often emphasised and punctuated by loud cracks as Dr Miller adjusts the patient’s spine*” (p. 315). Furthermore, Jamison [35] suggested that the working relationship has therapeutic elements, and the encounter could have an “intrinsic psychotherapeutic effect” because of this perception that change is happening: “*Formulation of a working diagnosis resulted from dynamic interaction between the patient and the practitioner, and this became the focus for immediate therapeutic intervention. Something was being done!*” (p. 97). Patients also confirmed the importance of this notion of change [39]: “*The only thing that would really make it [treatment] worthwhile is if I felt comforted from it, or I had a slight glimmer of hope that there’s going to be improvement. Otherwise, I don’t see the purpose in it*” (p. 11). Patients also noted that their idea of the change they desire to see is individual and subjective [39]: as one participant emphasised “*every patient here has their own story, so what is good for one person may not be good for another person*.” (p. 6). For chiropractors, this change may include not only the physical but also the psychological aspect of patients’ wellbeing [40]: “*We just try to change the mind-set right out of the get go*.” (p. 224). The change process may require patient education to facilitate negotiation and establish what the desired change can be and how to work collaboratively towards it. As one practitioner explained, the role of a chiropractor should prioritise patients’ needs and preferences [40]: “…*the focus should be revolving around their wants, not trying to subjugate their wants to my own…*” (p. 225).

### Chiropractic treatment as collaboration

On one hand, collaborative working was illustrated when a practitioner gives homework, offers education, provides explanations, and ensures that patients understand and agree. On the other hand, this theme also emphasises the role of patients’ active engagement in their treatment journey. According to the data, the relationship between the patient and the chiropractor is cooperative in nature [38]: “*Under chiropractic care, treatment is often negotiated with the patient, respecting the patient's autonomy.*” (p. 98). As the quote suggests, this theme also demonstrates the importance of negotiation.

Some patients in the qualitative component acknowledged their role in this change process [41]: “*…every time I go there, I get good advice, whether it’s ‘have you tried this’? Or with respect to changing your eating habits or some exercises…. ‘You know every time I go, it’s almost like I get a little nugget of information to get a shot to make the quality of my life better.*” (p. 4). Patients can have a more proactive approach [42]: “*The chiropractic treatments were amazing in that way. I learned about a new form of treatment and [another way to be proactive]*” (p. 159). In such cases, the change process is indeed negotiation between the patient and the chiropractor [42]: “*I trusted [the chiropractor] would understand, and he would always shift [his approach] based on whatever I was saying*” (p. 159). Conversely, some patients expect that their involvement in bringing about change will be minimal, and their practitioner is the one that will improve their circumstances [60]: “*I paid her to fix my back. I didn’t pay her to teach me how to fix my back*” (p. 224). Sadr and colleagues [43] noted in their study that “*only a few of the patients seemed to be very knowledgeable about their pregnancy and asked questions or challenged their chiropractors about various techniques or treatment*” (p. 4). It could be argued that educating the patient about their health and providing clear explanations about their treatment options may empower them to be more proactive in the negotiation. For example, Jamison [35] explored the establishment of WA in chiropractic and noted that although patient education was not a feature of every clinical encounter, it was “*a component of the total therapeutic regime and provided a foundation upon which patient could actively pursue shared therapeutic goals*” (p. 97). One of the chiropractors also emphasised the role of patient education [43]: “*I think the more knowledge they have [patients], the better they are… the woman who is going through the first pregnancy is very scared, hesitant, anxious and wants that kind of knowledge, and wants the practitioner to know what they’re going through and set their mind at ease.”* (p. 4). Overall, patients valued practitioners’ efforts to explain and teach them how to do things correctly [40] “*instead of just printing off some exercises*” (p. 225).

Regardless of participants’ beliefs about the level of their personal responsibility in their care process, the data revealed that patients would like to know what the plan is for bringing about change. Chiropractors considered that to be cooperative in nature, their approach should be honest and compassionate [44]: *“What I always say is that ‘We’re going to try to get you better, it might not be me. I might need help with other people. But the end result is that I’ll do everything I can to help you out’.*” (p. 101). Overall, qualitative data from chiropractors suggest that this collaboration includes communication, patient-centredness, mutual trust. The following themes will discuss in more detail these components of a collaborative working relationship.

### Communication

The third theme describes the communication between a patient and a chiropractor. First, example communication techniques serving different purposes were identified and are discussed in subtheme “Effective communication”. Second, potential conflicts are described as part of the clinical encounter in subtheme “Conflicts”.

### Effective communication

It was noted in one study [43] that *“communication between chiropractors and patients depended on the knowledge level of both parties”* (p. 4). Considering the importance of mutual understanding for collaboration as discussed above, one of the key communication goals should be clear explanation. A chiropractor should invest time to explain and to ensure that the patient has correctly interpreted the information [44]: “*clear and timely communication is an opportunity for chiropractors to understand patient expectations and assure patients that they are in a safe environment*” (p. 102). Practitioners acknowledged the importance of clear explanations in the negotiation process [40]: “*We try to really map it out in layman’s terms, this is why this is affected, and this is why if we can take the time to put in the work, it’s going to help. I think that’s been the most effective approach for sure, for adherence.”* (p. 225).

An explanation can be facilitated using non-verbal communication or analogies to illustrate a point [38]: for instance, “*…during his explanations, the D.C. often actively demonstrates the movement or procedure he wants his patients to practice, thus identifying with the role of the patient.*” (p. 97). This is useful for the patient in two ways: not only will they have a mental image of what the movement should look like, but also, they will feel more confident about doing it. Referring to Bordin’s formulation of WA [11], prioritising clear explanation as a communication goal can facilitate reaching a mutual agreement in relation to the goals of treatment and the tasks involved in the treatment plan.

Jamison [35] showed that practitioners may engage in both social and professional interaction with their patients: communication would be “*characterised by acceptance both of the patient as an individual and of their complaint as valid and worthy of diagnostic consideration and therapeutic intervention*” (p. 96). Similarly, Mior [45] discussed the qualitative data in their study by emphasising that *“the nature of the communication went beyond exploring the presenting complaint—the symptom—it focussed upon how their condition impacted upon the whole patient”* (p. 153)*.* In scenarios where the focus is the presenting complaint, communication may be entirely instrumentally oriented [38]: “*During an orthopaedic examination of a patient, the doctor is intent upon identifying the problem. A long battery of range of motion and pain tolerance tests are given. Therefore, most statements made are instrumentally oriented, usually consisting of directions, requests, and some information*.” (p. 97). Different communication techniques would be relevant if the purpose of communication is bonding on a more personal level. A chiropractor may use language in a person-centred manner [38] when they do not “*depersonalise a patient by referring to body parts with a definite article (e.g., 'the' neck looks fine today) but rather use a possessive pronoun (e.g., 'your' knee is swollen)*” (p. 105). Chiropractors recognised that the rapport can be further strengthened by comments of praise, encouragement, and reassurance [38]. Again, the role of non-verbal communication is key: a smile, handshake or eye-contact can create a friendly environment and the ability to read patients’ body language can inform a chiropractor on how to react accordingly [38, 44]. In other words, participants discussed the establishment of bond as an intentional goal of communication and recognised that this goal requires a particular set of communication techniques.

For example, active listening is of the utmost importance [44]: *“Uninterrupted listening provides an opportunity for patients to feel engaged and was described as a method of forming meaningful connection*.” (p. 101). Chiropractors mentioned that active listening requires time [41]: “*You try to direct the discussion as much as possible but give the patient the time to really explain what their experience has been, you know? I find that breaks down barriers really quickly and builds trust and confidence in a new person.*" (p. 5). Patients want to feel empathically understood and listened to [41]: “*We have a great relationship, and we talk a lot during the treatments, so I feel like my needs are being met*” (p. 5). Conversely, a condescending, disrespectful, disinterested approach, was described as a factor leading to dissatisfaction with care quality [46]. The role of active listening is also central when the goal of communication is shared decision-making or negotiation. In fact, one of the potential causes of conflicts between a patient and a chiropractor is misunderstanding.

### Conflicts

In the data, the definition of conflict included differences in opinion, tension, misunderstandings, failure to manage patients’ complaints, and unwillingness to refer the patient to other specialists. Oths [38] described example conflicts in the following context: “*Disagreements, tension, and passive and active antagonism tend to surface during critical points of the clinical encounter. Differences of opinion were sometimes voiced between doctor and patient. At times, this attested to the strength of their relationship when either showed s/he was not afraid to question or criticise the other's opinion*.” (p. 102). Differences in opinion were viewed as a test of the WA, which could be resolved via empathy, negotiation, and active listening.

While using communication techniques with a specific goal in mind can be effective, this can make a chiropractor less attentive to their patient’s comments when they focus on the task at hand. Non-attentiveness may also be the result of chiropractor’s beliefs: if a practitioner expects that the cause of pain is entirely biomechanical, then he or she may be less attentive to patient’s social and psychological concerns [37]: *“Dr. Miller explains that because of the power of the manual muscle test he does not need to get a detailed personal history from the patient: the body will tell him everything he needs to know. Consequently, much of the conversation is characterised by apparently unmotivated comments and questions about family, work, *etc.,* and general medical advice.*” (p. 308). Similarly, a patient described their negative experiences with chiropractors [45]: *“I don’t think that they showed the attention that they should have to the aches and pains that you were saying. They were almost focused on; well, this is what works and telling you that this is what the other doctors used to do, and it does work*.” (p. 157).

Occasions where non-medical details about a patient were remembered were considered beneficial for building trust. In contrast, beliefs about chiropractor’s sincerity could be undermined by their non-attentiveness [38]: “*this non-attentiveness seems to be the root of much of the tension occurring in exchanges*” (p. 103). Similarly, Mior [45] found that patients questioned the value of their treatment and the intentions of their chiropractor when too little time was spent building interpersonal relationships. Consultations where the practitioner only used manipulative therapy without any other therapies or did not prescribe exercises or lifestyle recommendations were perceived as negative experiences [45]. To avoid potential conflicts, chiropractors should pay attention to and seek to address potential signs of disagreement in a patient [38], which may be “*expressed as passive tension, primarily in the form of nervousness (usually with new patients), insecurity, overcaution, and dependency*” (p. 102). In cases of conflict [38], chiropractors’ negativity may be “*expressed as open antagonism, manifested by impatience or interrupting the patien*t” (p. 102). Conflicts are a likely part of the working relationship: attentiveness to the expectations of both parties involved should be prioritised.

### Patient-centredness as agreement on values, preferences and needs

Qualitative results from one of the studies [47] described patient-centredness as “*the quality of a chiropractor (and, importantly, all staff members) that demonstrates a provision of care that is respectful and responsive to the patient, and which is inclusive of the person’s values, preferences, and needs*” (p. 6). Often it is “*expected the chiropractor to demonstrate this same quality (patient-centredness) in their interactions*” (p. 6). Overall, the person-centeredness may facilitate collaboration during care and the this theme provides examples.

Some patients [47] considered that “*…the chiropractor should have personal knowledge of each patient as well as information about the history of their injury and his or her current medical conditions. Such personal knowledge should then be integrated into the evolving care of the individual patient*” (p. 6). Such personal knowledge might enable practitioners to better understand each patient and facilitate collaboration. Indeed, patient’s perception that their chiropractor does not understand them was identified as a barrier to exercise adherence: considering patients’ values, preferences, and needs may influence patients’ active engagement in their care [40]. Patient-centredness may also impact the establishment of a mutual bond [42]: ‘*Participants also noted that the chiropractors listened and “would understand” and “shift” in response to their concerns, a cornerstone to building a trusting relationship*’ (p. 149). While trust is a key interpersonal process underpinning this bond, there are other positive feelings which patients associate with chiropractors whose approach is patient-centred [41]: “*It’s easy to feel like you’re friends with those kinds of professionals*.” (p. 5). Interestingly, the findings revealed that person-centredness comes with its challenges. For instance, a chiropractor [40] shared that they have “*probably sent people for x-rays as peace of mind for the patient*” (p. 224). Some practitioners felt that patients’ previous experiences and beliefs may have negative impact on the change process [40]: “…*they still feel like they need or they want the adjustment … we do it if we need to… but we don’t just kind of cater to expectations or wants from previous experiences…*” (p. 224). A practitioner [43] noted: “*The bio-psycho-social model is very relevant too. Because they are not all coming to me from nice family units…”* (p. 5).

The following quote from one of the studies [45] provides a good summary of this theme: “*The majority of patients felt the chiropractic care they received was patient-centred. They interpreted this as being involved, informed, and participant in approving the care they received. They reported being an active participant in the decision-making process of their care and the chiropractor seemed respectful of the patients’ needs and concerns.”* (p. 157). The examples discussed in this theme reveal how patient-centred approach which treats the patient as an individual with needs, values and preferences can facilitate the formation of WA and its three components: agreement on the goals of care, agreement and collaboration on the treatment plan and the foundation of positive reciprocal feelings. The next theme examines one of the key interpersonal processes involved in this mutual bond- the trust between a chiropractor and a patient.

### Trust

One study exploring the therapeutic encounter revealed that trust has a specific role in the working relationship [37]: “*Dr. Miller’s fundamental claim is that he is a healer. By this claim to legitimacy, he asserts that he is a qualified and practicing authority in the healing arts, and potentially helpful to people who present to him. As such he is allowed to make certain kinds of statements and do certain things, patients are correct to consult him in illness and he is entitled to the respect and rights accorded healers*.” (p. 310). It was suggested that practitioners’ trustworthiness depends on the credibility of chiropractic in general, which is usually validated through scientific evidence, experience, and good reputation. If the role of the chiropractor is that of the change agent, there are standards that should be considered. For instance, the chiropractic profession in the United Kingdom (UK) is regulated by law: The Chiropractors Act 1994 provides statutory regulation, and the title 'chiropractor' is protected under this legislation [48]. However, there are normative expectations which are more subjective and are examples of contextual factors in general clinical encounters. For example, the title doctor, the white coat, the tidy office, the medical jargon, the framed diplomas, and certificates, are all instances of symbolic representations of credibility. It should be noted that such contextual factors may also impact patients’ perception of chiropractor’s trustworthiness. One study [37] described how “*for some patients a clean office and an air of professional decorum are indicative of professional propriety and trustworthiness*” (p. 310).

The notion of honesty was emphasised [44]: “*Participants suggested that a trusting relationship would be established more quickly if they admit to mistakes and acknowledge their own limitations, which sometimes resulted in a referral.*” (p. 101). As the following quote shows, referring patients to other healthcare professionals who can better address their needs may increase their trust in the chiropractor [44]: “*When I refer them out to another discipline, another chiropractor or something like that, that actually they trust me more than anything else*” (p. 103). Chiropractors also acknowledged that agreement on goals and tasks is key for the establishment of trust between them and their patients. As one participant [44] explained about his approach: “*I tell them at the very beginning that I will never do things by surprise. I will always explain a thing before I do it. You are always the boss, I’m not. This visit is about you not me*.” (p. 103). Considering that the process of building trust is unique for each working relationship, chiropractors also pointed out the role of non-verbal communication. First, patients’ nonverbal communication reveals their level of trust and comfort in each situation. Second, chiropractors use their own nonverbal communication to establish trust [44]: “*eye contact, firm handshake, knowing when and when not to touch somebody*” (p. 102).

#### Summary

In summary, the qualitative synthesis illustrated the nature and role of WA in the chiropractic encounter. The professional relationship between a chiropractor and a patient aims to be cooperative in nature, involving collaboration and effective communication which is characterised by active listening, clear explanations, and patient education. The findings revealed the importance of trust and ongoing negotiation of expectations of the treatment plan whose main goal is to bring a mutually agreed upon change to patients’ circumstances.

### Quantitative component

First, this section provides a summary of the included study designs and participants. More details about each study (participants, methods of data collection, and data analysis) are shown in Table 4 which can be found at the end of the document text file.

**Table 4:** Summary of studies included in the quantitative synthesis

Study reference number	Country	Participants	Methods of data collection	Methods of data analysis
[71]	United States (US)	343 Pregnant patients	Questionnaires	Descriptive statistics and the paired t test
[53]	Canada	69 Military personnel presenting for on-site chiropractic services	Questionnaire	Multivariable regression model
[34]	US	20 Patients with low back pain	Questionnaires	Student's t-test
[44]	Canada	6 Chiropractors licensed with the College of Chiropractors of British Columbia	Questionnaire	Descriptive statistics
[72]	US	20 Institutions provided a written copy of the Informed consent documents	Retrieving Informed Consent (IC) documents	IC were compared against a list of requirements
[73]	United Kingdom (UK)	509 Participants from four participating UK associations	Telephone survey	Pearson Chi-Square tests
[74]	Australia	153 Patients with chronic conditions	Questionnaires	Descriptive and summary statistics
[75]	US	1759 Adults in the United States population	Questionnaire	Descriptive and multivariate methods
[76]	US	66 Questionnaires were returned from new and established patients	Questionnaire	Not explicitly specified
[54]	US	400 Participants with chronic low back pain	Questionnaire	Path analysis
[77]	US	681 Patients randomized, 341 were assigned to the 2 chiropractic groups	Questionnaires at baseline and 2 weeks of treatment	Mixed linear modelling
[55]	US	Same trial as in the study of Hertzman-Miller et al. [77]	Questionnaires	Multiple logistic and linear regression modelling
[35]	Australia	144 Patients seeing chiropractors with practices in diverse locations	Questionnaires	Not explicitly specified
[36]	Australia	9 Chiropractors and 173 patients participated	Questionnaires	Descriptive statistics
[49]	The Netherlands	89 Chiropractors, 207 patient–chiropractor working relationships	Questionnaires	A one-way ANOVA (analysis of variance) test
[78]	US	72 Chiropractors who worked in 61 chiropractic practices	Questionnaire	Regression analysis
[55]	Canada	2597 Patients participating in a collaborative study	Questionnaires	A multiple linear regression model
[38]	US	A sample of 57 people, who made a total of 104 office visits between them	(1) Audiotape of all clinical interaction of the chiropractor for 8 days (2) patient questionnaires pertaining to satisfaction with care	(1) Data was content analysed using the modified Bales method of process analysis [69]. (2) descriptive statistics
[79]	UK	465 Practitioners of which 132 chiropractors (28%)	Questionnaires	Several multivariate analyses of variance (ANOVA)
[52]	US	541 New and returning chiropractic patients	Questionnaires	Multiple stepwise regression analysis
[80]	Sweden	30 Chiropractors and 336 patients from 17 private practices	Questionnaires	The Wilcoxon signed rank test
[81]	US	62 Chronic pain patients recruited from four chiropractic offices	Questionnaires	Series of multiple regression analyses
[41]	Canada	90 Participants were recruited from two private chiropractic clinics	Questionnaire	Descriptive statistics

#### Quantitative study characteristics

The 23 included papers were comprised of quantitative descriptive studies, randomised controlled trials and a study conducting analysis on documents. The quantitative components of mixed method studies used questionnaires. Most chiropractic patients presented with musculoskeletal problems. Two studies focused on women with migraine and pregnant women. Studies were conducted in the United States, Canada, Australia, the United Kingdom, the Netherlands, and Sweden..

#### How is working alliance measured in the chiropractic literature?

Table 5 which can be found at the end of the document text file illustrates how WA was explored either explicitly or implicitly and which measurement tools were used. This included validated tools designed to measure WA, surveys and questionnaires asking participants about their relationship with their chiropractor and/or assessing any of the three features of WA proposed by Bordin [11]. Only two studies measured WA explicitly [35, 49]. First, Jamison [35] used a mixed-method study to explore the perceptions and experiences of patients during care. The questionnaire designed for the quantitative component included, for example, items assessing patients’ perceptions of their psycho-emotional state before treatment and their expectations of their psycho-emotional state after treatment. Second, Lambers and Bolton [49] used patient and chiropractor versions of the “Werkalliantievragenlijst (WAV-12) [50], which is a shortened and revised version of the Working Alliance Inventory (WAI) [18, 50, 51]. Additional File 3: Table S2 provides a more detailed summary of how WA and its components were measured.

**Table 5:** Measuring working alliance (WA)

Study reference number	How was WA measured?	Which tool was used?
[71]	Specific items in the visit-specific satisfaction questionnaire implicitly measured WA	The RAND VSQ9 is a 9-item questionnaire adapted by the American Medical Group from the Visit Rating Questionnaire used in the RAND Medical Outcomes Study [82]
[53]	Specific items in the satisfaction questionnaire implicitly measured WA	The 27-item satisfaction questionnaire used for this study was adapted from the chiropractic satisfaction survey [52]
[34]	Specific items in the satisfaction questionnaire implicitly measured WA	Satisfaction questionnaire designed for this study
[44]	The study measured chiropractors’ perception of trust, which implicitly explored one of WA’s features (bond)	The survey was created following qualitative analysis to verify emerging themes related to chiropractors’ perceptions of trust
[72]	The study evaluated questions for informed consent, which implicitly measured two features of WA (agreement on goals and tasks)	A list of questions was developed by the principal authors based on what they believe an educated patient considering chiropractic management of low back pain would want to know before making an informed decision about their care and providing their informed consent
[73]	The survey assessed behaviour patterns’ of chiropractors’ (1) goal-setting, (2) reevaluating progress with their patients. and (3) the discussion of addressing lifestyle issues. This study implicitly explored two features of WA (agreement on goals and tasks)	Survey designed for this study
[74]	This study measured patient-centred care during consultation with practitioners, which implicitly explored two features of WA (agreement on goals and tasks)	Patient-Centred Care Scale and the Patient Assessment of Chronic Illness Care measure (The PACIC)
[75]	Specific items in the satisfaction questionnaire implicitly measured WA	A national telephone satisfaction survey
[76]	Specific items in the satisfaction questionnaire implicitly measured WA	A visit-specific questionnaire that included a set of 9 items adapted from the Group Health Association of America Visit-Specific Questionnaire
[54]	Authors explored the doctor–patient encounter (DPE), which implicitly measured WA	Satisfaction Questionnaire by Cherkin and colleagues [34]
[77]	Specific items in a satisfaction questionnaire implicitly measured WA	Satisfaction Questionnaire by Cherkin and colleagues [34]
[55]	This is the same trial as in the study of Hertzman-Miller and colleagues [77]	
[35]	Mixed method study explicitly exploring WA	The quantitative component used a closed-question questionnaire to assess the perceptions and experiences of patients
[36]	Exploring the congruence of patient–practitioner perceptions implicitly measured two features of WA (agreement on goals and tasks)	A patient questionnaire and a practitioner questionnaire
[49]	The study explicitly measured WA	The Werkalliantievragenlijst [49]
[78]	Exploring social communication skills of practicing chiropractors implicitly assessed aspects of WA	Riggio's Social Skills Inventory (SSI) [83–85]
[45]	The study evaluated key aspects of the care provided to patients, and some of the items assessed implicitly WA	The Primary Care Assessment Survey (PCAS)
[38]	The study explored WA implicitly via examining chiropractors’ communication and interaction patterns	All taped verbal dialogue between the practitioner and his patients was content analysed using the modified Bales method of process analysis [69]
[79]	The study investigated the attitudes to back pain using a recently developed and validated questionnaire. This implicitly explored two features of WA (agreement on goals and tasks)	The Attitudes to Back Pain Scale for musculoskeletal practitioners (ABS-mp)
[52]	Specific items in the satisfaction questionnaire implicitly measured WA	Satisfaction questionnaire
[80]	The study explored the expectations of new patients consulting a chiropractor and to evaluated differences and similarities in expectations between chiropractors and patients. This implicitly measured two features of WA (agreement on goals and tasks)	Questionnaires
[81]	Explored how attached or connected patients feel toward their chiropractor, which implicitly measured one of WA’s features (bond)	Physician–Patient Attachment Scale (PPAS) [86]
[41]	The study assessed patient-centred care in patients with chronic health conditions attending chiropractic practice. This study implicitly assessed two features of WA (agreement on goals and tasks)	A modified version of the Patient Assessment of Chronic Illness Care (PACIC) [87]

#### What is the effect of working alliance in chiropractic on clinical outcomes and patients’ satisfaction?

Eight studies measured patient satisfaction by using scales consisting of items which also assessed aspects of WA. For instance, a 27-item satisfaction questionnaire adapted from the chiropractic satisfaction survey [52] was used by Boudreau and colleagues to explore patient satisfaction associated with the introduction of chiropractic services within a military hospital [53]. Example item assessing aspects of WA included in this questionanire was “My chiropractor treated me with respect” which implicitly explored the bond [11] between a patient and their chiropractor [53]. While the findings of the quantitative component suggest that WA plays a role in patients’ subjective evaluation of their satisfaction with care, no studies measured the impact of WA on either clinical outcomes or patient satisfaction explicitly. Three studies examined the impact on clinical outcomes using tools which implicitly explored WA by measuring the impact of doctor–patient encounter (DPE) [54] and patient satisfaction [45, 55]. First, Haas end colleagues [54] measured low back pain (LBP) intensity via the Modified Von Korff pain scale [56] to evaluate the effects of the DPE via path analysis. The results revealed that DPE was a determinant of LBP at both follow-ups at 6 and 12 weeks [54]. Second, Mior [45] used a variable reflecting the patients’ subjective report of symptomatic improvement after receiving their treatment. He reported that the results of regression analyses showed that both high patient satisfaction and feeling much better following a treatment were positively associated with ratings of the chiropractor as a high performer on all the PCAS scales [57]. Next, Hurwitz and colleagues [55] explored the effects of patient satisfaction on subsequent changes in pain and disability among LBP patients with the 24-item Roland-Morris Disability Questionnaire Disability [58, 59], and remission from clinically meaningful pain and disability. Authors [55] found that higher satisfaction improved the odds of remission from clinically meaningful pain and disability at 6 weeks. It should be noted that these three studies measure constructs adjacent to WA and findings should be interpreted with caution. Table 6 which can be found at the end of the document text file shows more details of the results.

**Table 6:** The effect of working alliance on clinical outcomes and satisfaction

Study reference number	How did the study measure clinical outcomes?	Did the study measure satisfaction? If yes, how?	What did the study find?
[54]	Low back pain intensity, which was a primary study outcome, was assessed using the Modified Von Korff pain scale [56]. A path analysis was conducted to determine the effects of dose and doctor–patient encounter (DPE) on LBP intensity at the end of care (6 weeks) and primary end point (12 weeks)	No	The principal finding was that the DPE evaluated at the end of care and Spinal Manipulation Therapy (SMT) dose had similar effects on pain outcomes. DPE β = − 0.22 and − 0.15 and dose β = − 0.11 and − 0.12 for the six and 12-week pain outcomes, respectively. Patients’ perception of their practitioners’ enthusiasm and confidence related to the treatment process may impact the establishment of trust and respect, which underlies the bond between patients and their chiropractor
[55]	At every follow-up assessment: functional status was measured by repeat Roland-Morris Low-Back Disability [58, 59]. Questionnaires, pain status was measured by repeat numerical rating scales and scales of global pain improvement, and pain frequency measured by 6-point ordinal scale	Satisfaction with back care was measured on a 40-point scale and observed at 4 weeks following randomisation	Greater satisfaction increased the odds of remission from clinically meaningful pain and disability at 6 weeks (adjusted odds ratio (OR) for 10-point increase in satisfaction = 1.61, 95% confidence interval (CI) 0.99,2.68), but not at 6, 12, or 18 months (6 months: adjusted OR = 1.05, 95% CI 0.73, 1.52; 12 months: adjusted OR = 0.94, 95% CI 0.67, 1.32; 18 months: adjusted OR = 1.07; 95% CI 0.76, 1.50)
[45]	The “improved” variable reflected the patients’ subjective report of symptomatic improvement after receiving their treatment: it was dichotomised into two categories, either feeling much better or less than feeling much better after the treatment	Satisfaction was assessed on a 7-point Likert scale from 1 being ‘Completely satisfied, couldn’t be better’ to 7 being ‘Completely dissatisfied, couldn’t be worse’ from the question: “All things considered, how satisfied are you with your regular chiropractor.”	The results of regression analyses suggested that in general, patients who were completely satisfied with their overall chiropractic experience and felt much better following their treatment were positively associated with rating their chiropractor as a high performer on all the PCAS scales [57] when controlling for all other variables. For example, the adjusted odds ratio for the effect of being completely satisfied with your chiropractor when controlling for all other variables in the model was 9.97, suggesting a positive association between patients who are completely satisfied with their overall chiropractic experience and rating their chiropractor above the 75th percentile on the Trust in chiropractor scale. Similarly the variable improved was also positively associated with patients rating their chiropractor as a high performer in the Trust in chiropractor scale

The text in this table used phrasing as close as possible to the phrasing used by authors when reporting their studies

## Discussion

### Summary of findings

This review included thirty studies exploring aspects of the WA between a chiropractor and their patients to study its nature and role within the clinical encounter. The qualitative component highlights the importance of WA during the treatment process and emphasise the role of effective communication. For patients, the perception that their beliefs, values, and preferences have been attended to by the practitioner strengthens the WA and might facilitate treatment adherence. For chiropractors, patients’ previous experiences, unhealthy beliefs and unrealistic expectations can challenge the establishment of a collaborative working relationship. Most of the studies included in the thematic synthesis had good methodological quality. The qualitative findings of two mixed-method studies did not contribute substantially to the synthesis [34, 36]. This was due to the lack of details provided by the authors: it was unclear whether the findings were adequately derived from the data and the interpretation of results was not sufficiently substantiated by data. The quantitative component found only two studies explicitly investigating the WA between a chiropractor and a patient [35, 49]. The studies included in the narrative synthesis were appraised highly on most methodology criteria apart from the risk of non-response bias. However, this criterium did not impact the quality of the synthesis in regards to investigating how WA and its features have been measured in the literature. To our knowledge, no studies have explored explicitly the impact of WA on clinical outcomes and patient satisfaction.

### How do the integrated findings of this systematic review fit the wider literature?

Babatunde and colleagues conducted a scoping review of the literature studying WA across physiotherapy and occupational therapy [60]. They found that WA has been explored only to a limited extent in the rehabilitation literature and suggested that future research should prioritise clear conceptualisation of the construct [60]. The findings from our review of WA in chiropractic illustrate the potential which the construct and Bordin’s theory [11] have to explain how the relationship between a chiropractor and a patient influences treatment outcomes through psychosocial pathways.

The qualitative synthesis suggests that chiropractic care can be considered as a prolonged change process in which collaborative working relationships between patients and chiropractors are especially important. At the start of care, a patient who seeks help to change their circumstances and a chiropractor whose role is that of the change-agent begin a treatment journey together. Strong WA can facilitate the change process ensuring its cooperative nature: our synthesis related patients’ and chiropractors’ experiences of therapeutic relationships to the role of the three features proposed by Bordin [11]: agreement on the goals of care, collaboration on the treatment plan, and the establishment of a bond. For instance, the quantitative synthesis shows there might be a mismatch between patients’ and chiropractors’ expectations about what patients’ role in the treatment journey should be [36]. Second, agreement on the treatment plan should be reached: comprehensive instructions of what is required from the patient can promote a proactive approach to care by shaping patients’ beliefs about their own capability (self-efficacy) to adhere to the treatment plan [61]. The qualitative synthesis suggests that patient education regarding the treatment plan is crucial: patients appreciate when they understand how the recommended tasks can lead to the desired outcome. In line with a patient-centred approach, which is a paradigm of chiropractic [62], strong WA involves shared decision making throughout care and focus on individuals’ preferences, needs and values. The current findings show that there could be a discrepancy in patients’ and chiropractors’ perceptions of the level of collaboration between them and their chiropractor [49]. Practitioners should continuously try to collaborate with their patients to ensure that agreement on treatment plan and goals of care is established during all stages of this journey [49]. The third key factor impacting this change process is the bond underpinned by reciprocal positive feelings of respect and trust. For example, the qualitative synthesis illustrates that such a bond is key when chiropractors aim to identify and change patients’ unhealthy beliefs and behaviours which might be compromising their treatment progress [40].

This notion of considering chiropractic care as a change process is in line with the dynamic model of treatment perceptions [63]. The model was developed using grounded theory analysis to analyse interview data from patients receiving chiropractic treatment for back pain and was then tested using interview data from people undertaking exercise therapy for dizziness [63]. According to this model, at the start of this journey, patients have their abstract treatment perceptions which reflect their personal beliefs, values, norms, as well as their illness representations and health-related beliefs [63]. These newly modified treatment perceptions are influenced by patients’ pre-existing expectations, their interactions with the practitioner, perceived changes in symptoms, perceived chiropractor’s competence, and the overall care experience [63]. Similarly, the current literature review reveals that the treatment journey potentially leads to both physical changes and psychological changes which are a consequence of the concrete experiences during clinical encounters: patients' abstract representations of the treatment are adjusted accordingly [63]. For example, the qualitative component illustrates that the interactions between a chiropractor and a patient may facilitate exercise adherence or change patients’ mindset [40]. The qualitative component also shows that patients seek validation that change is indeed occurring throughout the treatment journey. The findings also suggest that this validation is subjective: for example, a symbol of change could be the cracks during spinal manipulation, the gained knowledge, the reduced pain, or the improvement in physical function.

The themes generated by the thematic synthesis of this review are in line with the findings of a concept analysis which explored WA within physiotherapy literature [64]. Authors concluded that the conceptualisation of WA as outlined within their themes share similarities with Bordin’s conceptualisation [64]. The themes revealed the importance of the attention which a physiotherapist paid to the patient, the shared therapeutic journey aiming to assist the patient from being dependent to independent, the sense of safety allowing patients to unfold themselves both physically and psychologically, and the role of communication which acted as a catalyst in operationalising the WA in a physiotherapy context [64]. Also, a qualitative systematic review and meta-synthesis investigated patients’ and physical therapists’ perceptions of factors that impact their mutual relationship [65]. The review found four themes which influenced patient-therapist interactions: (1) physical therapists’ interpersonal and communication skills; (2) physical therapists’ practical skills; (3) individualised patient-centred care; and (4) organisational and environmental factors. In correspondence with our findings, the authors noted that patients appreciated a physical therapist with good listening skills and empathetic friendly demeanour [65]. Furthermore, a systematic review of the literature studied the impact of WA in physical therapy for chronic musculoskeletal pain and evidence from three studies suggested that strong WA may improve pain outcomes [66].

The quantitative component of our systematic review included only two studies measuring explicitly WA. Most studies explored adjacent constructs, for example, communication, patient satisfaction, and dimensions of person-centred care. For instance, questionnaires which measured patient satisfaction contain items focusing on contextual factors inherent in WA. Despite that the scales measuring these adjacent constructs include items enquiring about the chiropractor–patient bond, the collaboration on tasks and/or the agreement on goals, they have less explanatory power than a scale specifically designed to measure WA. This review provides some initial evidence for the role of WA within the chiropractic clinical encounter. Research explicitly exploring this construct should measure its direct and mediated effects on clinical outcomes and patient satisfaction. For example, Bishop and colleagues conducted a large prospective cohort study, and their findings emphasised the role of WA and its three features [11] as a contextual predictor of back-related disability over time in physiotherapy, osteopathy, and acupuncture [5]. They suggested that strong WA has the potential to increase patient self-efficacy for coping with pain and to lessen the perceived threat of pain and alleviate psychosocial distress [5]. The authors also proposed that it could be useful to develop and then trial post-qualification training for practitioners to enable them to utilise the clinical value of WA in their practice [5]. Considering the foundational ideas of chiropractic emphasise the idea that the body is a self-healing mechanism [67], and the evidentially supported notion that self-healing can be triggered by contextual factors intrinsic to the patient–practitioner relationship, the role of WA in chiropractic consultations should be better understood [15, 68].

### Implications of findings

The qualitative component illustrates that Bordin’s formulation of WA [11] has the potential to explain the impact of chiropractor–patient relationship on patient outcomes. The quantitative component shows that even though the construct is relevant to the chiropractic discipline, there is a limited amount of research focused on WA. The findings from this review emphasise the value of measuring the direct and mediated effects of WA between a patient and a chiropractor on patient outcomes. One sensible next step would be to conduct primary research exploring the potential psychosocial pathways through which WA impacts clinical outcomes and patient satisfaction [5].

### Strengths and limitations

This research project synthesised a diverse body of evidence on the topic of WA using data from quantitative, qualitative, and mixed method studies. This type of systematic review provided a nuanced understanding of such a multifaceted phenomenon and was appropriate for the explorative objective of this study. However, there were not enough homogenous studies measuring the impact of WA on patients’ satisfaction and clinical outcomes to conduct a meta-analysis. Narrative synthesis provides more limited information for healthcare decision making than meta-analysis [21, 29]. Most studies measured WA implicitly using tools designed for other purposes, suggesting the need for more research on the topic in the context of the chiropractic profession. Furthermore, due to the lack of exact definition of the concept of WA in the literature, it is possible that studies exploring the WA between a chiropractor and a patient using conceptualisation different to the one offered by Bordin [11] were not included in the results. Therefore, given that the search strategy was based on this conceptualisation, it likely influenced the qualitative findings. Nevertheless, the qualitative data informed the thematic synthesis and shaped the generated themes.

## Conclusion

WA has been studied to a limited extent within the chiropractic discipline. The nature of WA is best understood if chiropractic care is viewed as a change process in which a patient aims to improve their circumstances by seeking help from their practitioner whose role is that of the change agent. Strong WA requires ongoing negotiation of treatment goals and expectations alongside collaboration on a mutually agreed upon treatment plan. These processes of negotiation and collaboration are facilitated by, and may in turn strengthen, interpersonal bonds involving trust and mutual respect. Bordin’s formulation of WA [11] has the potential to improve our understanding of chiropractor–patient relationships by providing a conceptual framework for thinking about the nature of the therapeutic relationship and how it can impact clinical outcomes through psychosocial pathways. Further primary research is needed to establish the nature, appropriate measurement, and consequences of WA in chiropractic care.

## Supplementary Information

**Additional file 1. Figure S1.** PRISMA 2020 flow diagram.

**Additional file 2. Table S1.** Quality Appraisal.

**Additional file 3. Table S2.** How was the Working Alliance measured?

## Data Availability

The extracted data tables and the codebook from the thematic synthesis supporting the conclusions of this article will be available in the University of Southampton Institutional Research Repository. Making the data available is currently in process and in the meantime, data is available on request from the corresponding author (DI).

## Abbreviations

IC: Informed Consent
JBI: Joanna Briggs Institute
MMAT: Mixed Methods Appraisal Tool
PPAS: Physician–Patient Attachment Scale
PICo: Population, Phenomena of Interest, Context
PRISMA statement: Preferred Reporting Items for Systematic Reviews and Meta-analyses
PCAS: Primary Care Assessment Survey
SSI: Riggio's Social Skills Inventory
UK: United Kingdom
US: United States
WAV-12: Werkalliantievragenlijst
WA: Working Alliance
WAI: Working Alliance Inventory
