# Supplementary material for: Mixed methods systematic review of the literature base exploring working alliance in the chiropractic profession
Source: Chiropr Man Therap. 2022 Sep 2;30:35. doi: 10.1186/s12998-022-00442-4 (PMC9438171; doi:10.1186/s12998-022-00442-4)
Supplement: Supplementary file 1 — Additional file 1. Figure S1. PRISMA 2020 flow diagram. [file 12998_2022_442_MOESM1_ESM.pdf]

Figure 1: **PRISMA 2020 flow diagram**

i

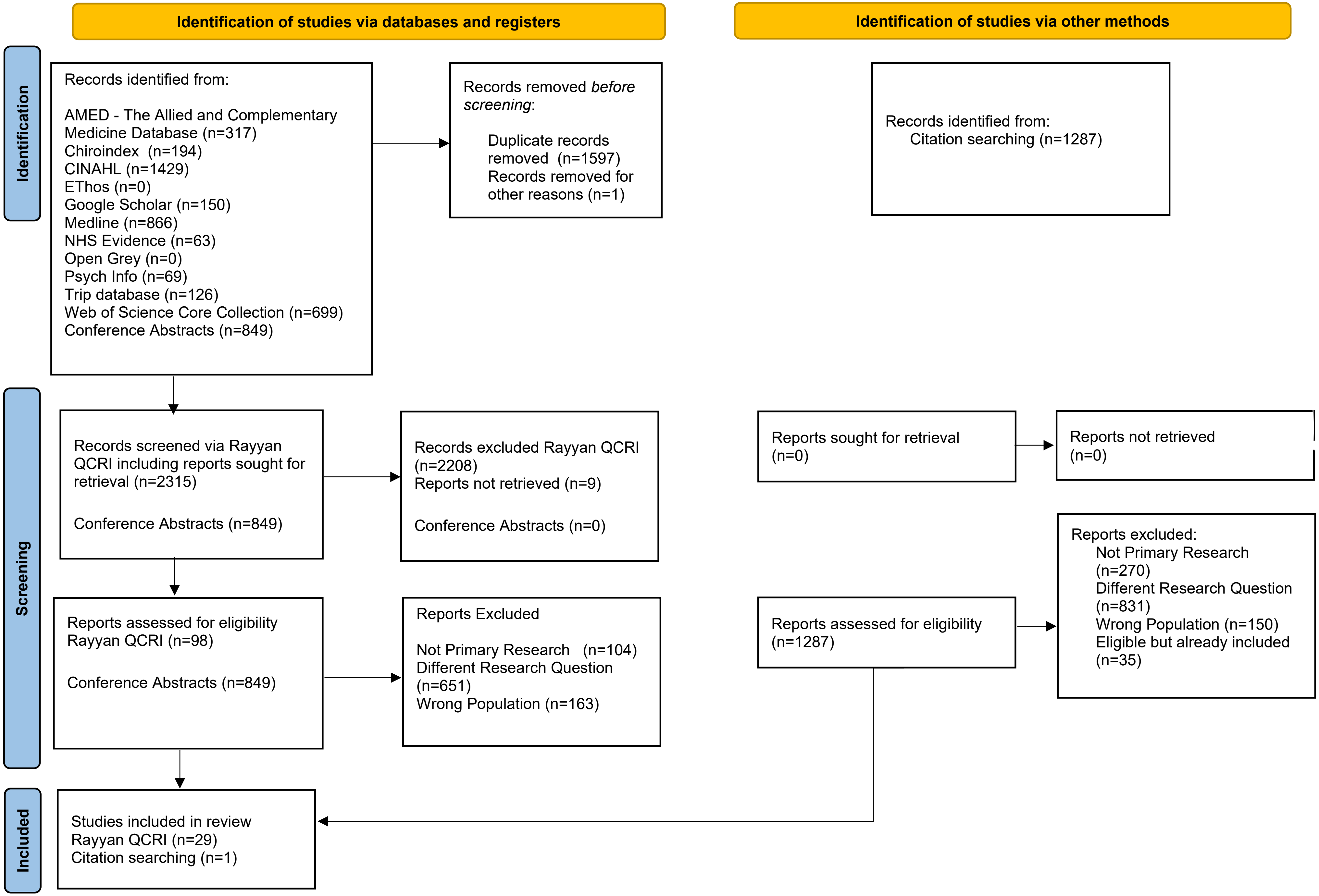

Figure 1: **PRISMA 2020 flow diagram**

---

[i] The screening of Conference Abstracts and Citation searching was conducted manually instead of using Rayyan.

From: Page MJ, McKenzie JE, Bossuyt PM, Boutron I, Hoffmann TC, Mulrow CD, et al. The PRISMA 2020 statement: an updated guideline for reporting systematic reviews. BMJ 2021;372:n71. doi: 10.1136/bmj.n71. For more information, visit: http://www.prisma-statement.org/
