# Supplementary material for: Mixed methods systematic review of the literature base exploring working alliance in the chiropractic profession
Source: Chiropr Man Therap. 2022 Sep 2;30:35. doi: 10.1186/s12998-022-00442-4 (PMC9438171; doi:10.1186/s12998-022-00442-4)
Supplement: Supplementary file 3 — Additional file 3. Table S2. How was the Working Alliance measured? [file 12998_2022_442_MOESM3_ESM.docx]

**Table S2: How was the Working Alliance measured?**

| **Study Reference Number** | **Is Working Alliance measured implicitly or explicitly?** | **Which Bordin’s Working Alliance feature is measured?** | **Which tool was used?** | **Example Items** |
| --- | --- | --- | --- | --- |
| (1)* | Implicitly | Agreement on tasks and the bond. | The RAND VSQ9 (2). | - Explanation of what was done to you? - Technical skills (thoroughness, carefulness, and competence) of the physician/ healthcare professional you saw? - The personal manner (courtesy, respect, sensitivity, and friendliness) of the person you saw? |
| **(3)*** | Implicitly | All three features. | The 27-item satisfaction questionnaire used for this study was adapted from the chiropractic satisfaction survey **(4).** | - All of my questions were answered by my chiropractor. - My chiropractor did his best to keep me from worrying about my problem. - My chiropractor was interested in all my health problems. - My chiropractor treated me with respect and concern. - My chiropractor made me feel foolish. - My chiropractor didn’t give me suggestions on what I could do to help my problem. - My chiropractor gave me advice on how to prevent health problems from occurring. - I think that my chiropractor should have spent more time. - My chiropractor acted as though I was important. - I feel I had to see my chiropractor more than I should have. - My chiropractor was very careful to check everything when examining me. |
| **(4)*** | Implicitly | All three features. | Satisfaction questionnaire. | - My doctor was interested in all my health problems. - All of my questions were answered by my doctor. - My doctor treated me with respect and concern. - My doctor did not give me suggestions on what I could do to help my problem. |
| **(5)*** | Implicitly | All three features. | Satisfaction questionnaire designed for this study | - My doctor seemed to believe my pain was real. - Provider gave instructions on back exercise. - Strongly agree I knew what to do to take care of my back after my visit. - Provider seemed confident that the treatment s/he recommended would work. - Provider understood my concerns about the cause of my pain. |
| **(6)** | Implicitly | Bond. | The survey was created following qualitative analysis to verify emerging themes related to chiropractors’ perceptions of trust. | To what extent do you agree with the following statements:   - Chiropractors who are honest are more likely to form trust with their patients. - My patients are more likely to trust me if I’m authentic. - Understanding my patient’s body language can help me understand if they trust me. - I believe that clear communication is a way to build trust with patients. |
| **(7)** | Implicitly | Agreement on goals and tasks. | A list of questions was developed by the principal authors based on what they believe an educated patient considering chiropractic management of low back pain would want to know before making an informed decision about their care and providing their informed consent. | Example questions:   - What could go wrong with this treatment? - What are the specific goals of this treatment? |
| **(8)** | Implicitly | Agreement on goals and tasks. | Survey designed for this study to explore behaviour patterns of chiropractors. | The items were organised into categories, namely, nutrition, physical activity, psychosocial well-being, smoking and alcohol consumption. Behaviour patterns such as goal-setting, revaluating progress with patients, and discussing lifestyle issues were examined. |
| **(9)** | Implicitly | Agreement on goals and tasks. | Patient-Centred Care Scale and the Patient Assessment of Chronic Illness Care measure (The PACIC). | - Asked to talk about my goals in caring for my condition. - Helped to set specific goals to improve my eating or exercise. - Asked for my ideas when we made a treatment plan. - Given choices about treatment to think about. |
| **(10)*** | Implicitly | All three features. | A national telephone satisfaction survey. | Satisfaction with particular aspects of the chiropractor:   - Provides good advice about staying healthy and preventing illness. - Provides effective advice for routine problems and illnesses. - Effective in diagnosing serious problems and recommending action. - Explains health problems and choices available. - Willing to refer to appropriate specialists when necessary. - Orders and interprets appropriate laboratory tests. - Concerned about me as a person and my overall health.     Extent of personal involvement in health care decision making:   - Participate in health care decision making but rely heavily on provider. - Responsible for own health decisions, but value provider’s advice. - Generally, make own health care decisions. |
| **(11)*** | Implicitly | All three features. | A visit-specific questionnaire that included a set of 9 items adapted from the Group Health Association of America Visit-Specific Questionnaire. | Patients were asked to indicate how satisfied they are by answering questions relating to time spent with the provider, explanation of what was done, technical skills of the provider, personal manner of the provider, and the visit overall. Examples were not provided. |
| **(12)** | Implicitly | All three features. | Satisfaction Questionnaire by Cherkin and colleagues **(5).** | Measures of the doctor-patient encounter included patient perception of chiropractor enthusiasm for care, comfort treating low back pain, confidence in care success, and adequate time spent with the patient. An example question was “My doctor seemed enthusiastic about my treatment program”. |
| **(13)*** | Implicitly | All three features. | The study measured satisfaction using the questionnaire by Cherkin and colleagues (5). Also, the authors measured the provision of self-care advice and if the chiropractor explained the treatment plan. | - An example item relevant to WA from the Information subscale is "The doctor gave me enough information about the cause of my pain", while the Caring subscale contained items such as "My doctor seemed to believe my pain was real". - Self-care advice was measured by summing how many of 10 specified types of advice the subject reported having received from their primary provider. - The explanation-of-treatment variable was the subject’s answer (yes or no) to the question “Did your provider explain your low back pain treatment plan (for example, did he or she tell you how often you should schedule visits)?”. |
| **(14)*** | This is the same trial as in the study of Hertzman-Miller and colleagues **(13).** | | | |
| **(15)** | Explicitly. | All three features. | The quantitative component used a closed-question questionnaire to assess the perceptions and experiences of patients. | - Patients could circle items such "overwhelmed, down, understood, trapped, hopeful, etc.'' to describe their current and expected feelings relating to chiropractors' care. - They could also circle statements describing their opinion on items such as "I feel that my chiropractor is interested in". For this item, the options were "my problem” or “me, the person” etc. - Regarding suggestions of how to improve the chiropractic care, patients could indicate if they wanted their practitioner to spend more time "listening to their concerns” etc. |
| **(16)** | Implicitly | Agreement on goals and tasks. | A patient questionnaire and a practitioner questionnaire. | The items assessed the concurrence of patient-practitioner perceptions with respect to the patient's stress levels, the importance of injury as a causative factor in the presenting symptom and the responsibility the patient should take “in getting themselves well.” Examples were not provided. |
| **(17)** | Explicitly | All three features. | The Werkalliantievragenlijst ([49](#_ENREF_49)). | Items in the questionnaire were in Dutch. Each questionnaire consisted of twelve statements, reflecting patients’ experiences with respect to collaboration in reaching agreement on treatment goals (goal dimension), agreement on treatment strategies (task dimension) and on the existence of an affective bond (bond dimension). |
| **(18)** | Implicitly. | Bond. | Riggio's Social Skills Inventory (SSI) (19-21). | The inventory used recognises three basic types of skills in communication: expressivity, sensitivity, and control. These skills operate in two realms: the nonverbal and the verbal domain. An example scale of SSI is Emotional Sensitivity scale which measures the skills in receiving and interpreting the nonverbal communications of others with items such as “People often tell me that I am a sensitive and understanding person.” |
| **(22)** | Implicitly | All three features. | The Primary Care Assessment Survey (PCAS). | The PCAS subscales relevant to WA were 1) Knowledge of patient, 2) Communication, 3) Interpersonal treatment, and 4) Trust. Example item is  “Thinking about the personal aspects of the care you receive from your regular chiropractor, how would you rate the following” and then followed by “Chiropractor’s caring and concern for you”. |
| **(23)** | Implicitly | All three features. | All taped verbal dialogue between the practitioner and his patients was content analysed using the modified Bales method of process analysis (24). | The speech categories were combined to form three mutually exclusive indices: '   - “Positive Affect” consists of statements characterised by positive feeling or emotion and showing solidarity or tension release. - “Information Exchange” includes giving or asking for opinion, and giving or asking for orientation, consisting respectively of task- oriented interpretive or factual statements. - “Negative Affect” index subsumes all statements coded as showing disagreement, tension, or antagonism. |
| **(25)** | Implicitly | Agreement on goals and tasks. | The Attitudes to Back Pain Scale for musculoskeletal practitioners (ABS-mp) | - I explore the psychological problems that my patient is facing. - I often find myself providing psychological support to patients. - It is essential that I know about my patients' psychological difficulties. |
| **(26)** | Implicitly | Agreement on goals and tasks. | Questionnaires. | - “I expect that the chiropractor will explain what is wrong” - for patients - “I expect to explain to the patient what the problem is” - for chiropractors. |
| **(27)** | Implicitly | Bond. | Physician-Patient Attachment Scale (PPAS) (28). | - My physician listens to what I have to say. - If my physician knows something is bothering me, they ask me about it. - Talking over my problems with my physician makes me feel ashamed or foolish. |
| **(29)** | Implicitly | Agreement on goals and tasks. | A modified version of the Patient Assessment of Chronic Illness Care (PACIC)(30). | - Does your chiropractor help you set specific goals that you can work on? Was this done together? - What goals did you create with respect to diet or exercise? - Were these goals written down as part of a treatment plan? |

*Studies exploring patient satisfaction.

1. Alcantara J, Ohm J, Alcantara J. The use of PROMIS and the RAND VSQ9 in chiropractic patients receiving care with the Webster Technique. Complementary therapies in clinical practice. 2016;23:110-6.

2. Health R. 36-Item Short Form Survey (SF-36) <https://www.rand.org/health-care/surveys_tools/mos/36-item-short-form.html> [

3. Boudreau LA, Busse JW, McBride G. Chiropractic services in the Canadian Armed Forces: a pilot project. Military medicine. 2006;171(6):572-6.

4. Sawyer C, Kassak K. Patient satisfaction with chiropractic care. Journal of Manipulative and Physiological Therapeutics. 1993;16(1):25-32.

5. Cherkin DC, MacCornack FA. Patient evaluations of low back pain care from family physicians and chiropractors. Western Journal of Medicine. 1989;150(3):351.

6. Connell G, Bainbridge L. Understanding how chiropractors build trust with patients: a mixed-methods study. The Journal of the Canadian Chiropractic Association. 2020;64(2):97.

7. Dagenais S, Haldeman S. Shared decision making through informed consent in chiropractic management of low back pain. Journal of manipulative and physiological therapeutics. 2012;35(3):216-26.

8. Fikar PE, Edlund KA, Newell D. Current preventative and health promotional care offered to patients by chiropractors in the United Kingdom: a survey. Chiropractic & manual therapies. 2015;23(1):1-7.

9. Foley H, Steel A, Adams J. Perceptions of person-centred care amongst individuals with chronic conditions who consult complementary medicine practitioners. Complementary Therapies in Medicine. 2020;52:102518.

10. Gaumer G. Factors associated with patient satisfaction with chiropractic care: survey and review of the literature. Journal of manipulative and physiological therapeutics. 2006;29(6):455-62.

11. Gemmell HA, Hayes BM. Patient satisfaction with chiropractic physicians in an independent physicians' association. Journal of manipulative and physiological therapeutics. 2001;24(9):556-9.

12. Haas M, Vavrek D, Neradilek MB, Polissar N. A path analysis of the effects of the doctor-patient encounter and expectancy in an open-label randomized trial of spinal manipulation for the care of low back pain. BMC complementary and alternative medicine. 2014;14(1):1-11.

13. Hertzman-Miller RP, Morgenstern H, Hurwitz EL, Yu F, Adams AH, Harber P, et al. Comparing the satisfaction of low back pain patients randomized to receive medical or chiropractic care: results from the UCLA low-back pain study. American Journal of Public Health. 2002;92(10):1628-33.

14. Hurwitz EL, Morgenstern H, Yu F. Satisfaction as a predictor of clinical outcomes among chiropractic and medical patients enrolled in the UCLA low back pain study. Spine. 2005;30(19):2121-8.

15. Jamison J. The chiropractic consultation: establishing a therapeutic alliance. CHIROPRACTIC JOURNAL OF AUSTRALIA. 1996;26:94-9.

16. Jamison JR. Patient-practitioner perceptions: can chiropractors assume congruence? Journal of Manipulative and Physiological Therapeutics. 2000;23(6):409-13.

17. Lambers NM, Bolton JE. Perceptions of the quality of the therapeutic alliance in chiropractic care in the Netherlands: a cross-sectional survey. Chiropractic & manual therapies. 2016;24(1):1-11.

18. Marchiori DM, Henkin AB, Hawk C. Social communication skills of chiropractors: implications for professional practice. Journal of manipulative and physiological therapeutics. 2008;31(9):682-9.

19. Riggio RE. Assessment of basic social skills. Journal of Personality and social Psychology. 1986;51(3):649.

20. Riggio RE. The Social Skills Inventory (SSI): measuring nonverbal and social skills. The sourcebook of nonverbal measures: Going beyond words. 2005:25-33.

21. Riggio RE, Riggio HR, Salinas C, Cole EJ. The role of social and emotional communication skills in leader emergence and effectiveness. Group Dynamics: Theory, Research, and Practice. 2003;7(2):83.

22. Mior SA. Patients' Perceptions of the Primary Care Characteristics in a Model of Interprofessional Patient-centred Collaboration between Chiropractors and Physicians 2010.

23. Oths K. Communication in a chiropractic clinic: how a DC treats his patients. Culture, medicine and psychiatry. 1994;18(1):83-113.

24. Bales R. Interaction process analysis: a method for the study of small groups. 203. University of Chicago Press, Chicago; 1976.

25. Pincus T, Foster NE, Vogel S, Santos R, Breen A, Underwood M. Attitudes to back pain amongst musculoskeletal practitioners: a comparison of professional groups and practice settings using the ABS-mp. Manual therapy. 2007;12(2):167-75.

26. Sigrell H. Expectations of chiropractic treatment: what are the expectations of new patients consulting a chiropractor, and do chiropractors and patients have similar expectations? Journal of manipulative and physiological therapeutics. 2002;25(5):300-5.

27. Sims WD. Relationship of General Adult Attachment and Physician-patient Attachment with Experience and Expression of Anger Among Chronic Pain Patients: Oklahoma State University; 2009.

28. Winterowd Sa. Physician-patient attachment scale. . [Unpublished modified version of the Inventory of Parent and Peer Attachment.]. In press 2007.

29. Stuber KJ, Langweiler M, Mior S, McCarthy PW. A pilot study assessing patient-centred care in patients with chronic health conditions attending chiropractic practice. Complementary therapies in medicine. 2018;39:1-7.

30. Glasgow RE, Wagner EH, Schaefer J, Mahoney LD, Reid RJ, Greene SM. Development and validation of the patient assessment of chronic illness care (PACIC). Medical care. 2005:436-44.
